# Ultra‐Stretchable Kirigami Piezo‐Metamaterials for Sensing Coupled Large Deformations

**DOI:** 10.1002/advs.202303674

**Published:** 2023-12-03

**Authors:** Luqin Hong, Hao Zhang, Tobias Kraus, Pengcheng Jiao

**Affiliations:** ^1^ Ocean College Zhejiang University Zhoushan 316021 China; ^2^ Engineering Research Center of Oceanic Sensing Technology and Equipment Zhejiang University Ministry of Education China; ^3^ INM‐Leibniz Institute for New Materials 66123 Saarbrücken Germany; ^4^ Saarland University, Colloid and Interface Chemistry 66123 Saarbrücken Germany; ^5^ Shandong Institute of Advanced Technology Jinan 250000 China

**Keywords:** active sensing, bistable mechanoelectrical response, coupled large deformations, kirigami piezo‐metamaterials (KPM)

## Abstract

Mechanical metamaterials are known for their prominent mechanical characteristics such as programmable deformation that are due to periodic microstructures. Recent research trends have shifted to utilizing mechanical metamaterials as structural substrates to integrate with functional materials for advanced functionalities beyond mechanical, such as active sensing. This study reports on the ultra‐stretchable kirigami piezo‐metamaterials (KPM) for sensing coupled large deformations caused by in‐ and out‐of‐plane displacements using the lead zirconate titanate (PZT) and barium titanate (BaTiO_3_) composite films. The KPM are fabricated by uniformly compounding and polarizing piezoelectric particles (i.e., PZT and BaTiO_3_) in silicon rubber and structured by cutting the piezoelectric rubbery films into ligaments. Characterizes the electrical properties of the KPM and investigates the bistable mechanical response under the coupled large deformations with the stretching ratio up to 200% strains. Finally, the PZT KPM sensors are integrated into wireless sensing systems for the detection of vehicle tire bulge, and the non‐toxic BaTiO_3_ KPM are applied for human posture monitoring. The reported kirigami piezo‐metamaterials open an exciting venue for the control and manipulation of mechanically functional metamaterials for active sensing under complex deformation scenarios in many applications.

## Graphical abstract

Kirigami piezo‐metamaterials (KPM) are developed for sensing coupled large deformations caused by in‐ and out‐of‐plane displacements. The KPM sensors are integrated in the wireless sensing systems to human posture and vehicle tire bulge, which open an exciting venue for the control and manipulation of mechanically functional metamaterials for active sensing under complex deformation scenarios in many applications.

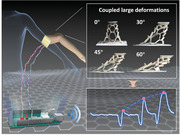

## 1 Introduction

Mechanical metamaterials have been reported with remarkable mechanical responses such as ultra‐lightness and ultra‐stiffness,^[^ ^1^, ^2^ ^]^ programmable deformation,^[^ ^3^, ^4^, ^5^ ^]^ tunable response,^[^ ^6^, ^7^, ^8^ ^]^ etc., mainly due to the periodic nature of their architected microstructures.^[^ ^9^, ^10^, ^11^ ^]^ Kirigami metamaterials take advantage of cutting plane structures to obtain tailorable stretchability for tunable mechanical properties,^[^ ^12^ ^]^ which have been conceived as a new class of easily adjustable and deformable frameworks for a variety of stretchable engineering applications, including energy harvesting,^[^ ^13^ ^]^ advanced sensing,^[^ ^14^ ^]^ self‐actuated response,^[^ ^15^ ^]^ etc. Recent research is aimed at utilizing kirigami metamaterials as structural substrates for the functionality beyond the mechanical domain, such as mechanoelectrical response in piezo‐metamaterials,^[^ ^16^, ^17^, ^18^ ^]^ tribo‐metamaterials,^[^ ^19^, ^20^, ^21^ ^]^ and other metamaterials with electrical functionality.^[^ ^22^, ^23^, ^24^ ^]^

Piezoelectric materials convert external mechanical excitations (e.g., applied forces or displacement) into electrical responses (e.g., voltage).^[^ ^25^, ^26^, ^27^ ^]^ Piezoelectricity has been extensively used for sensing different types of forces and deformations.^[^ ^28^ ^]^ In contrast to piezoresistive materials, piezoelectric materials can be independently operated to convert mechanical excitations into electrical signals without the severe requirement for external electrical sources.^[^ ^29^ ^]^ However, the existing piezoelectric materials are typically limited by their relatively inadequate stretchability.^[^ ^35^ ^]^ Previous studies have mainly focused on soft piezoelectric materials based on the flexible substrate.^[^ ^30^, ^31^ ^]^ More recently, studies have compounded rigid piezoelectric particles into flexible polymers and polarized them to obtain stretchable electronics that perform well with elastic characteristics under large deformations.^[^ ^32^, ^33^, ^34^ ^]^ To further improve the flexibility while achieving mechanoelectrical response tunability, recent studies have integrated piezoelectric materials with kirigami metamaterials to trigger the electrical materials in a more effective manner under various deformations such as in‐plane stretching,^[^ ^35^ ^]^ out‐of‐plane bending,^[^ ^36^ ^]^ wrinkling,^[^ ^37^ ^]^ etc. However, little has been done to tailor the kirigami design of the piezoelectric materials for sensing coupled large in‐ and out‐of‐plane deformations in complex application scenarios.

Here, we bridge this research gap and report on the ultra‐stretchable kirigami piezo‐metamaterials (KPM) that are created by cutting the lead zirconate titanate (PZT) and barium titanate (BaTiO_3_) rubbery films into kirigami metamaterials to sense coupled large deformations with up to 200% strains for the applications of, for example, vehicle tire bulge or human elbow posture monitoring. The KPM takes advantage of the mechanical stretchability of kirigami metamaterials and expands the piezoelectric response of elastically piezoelectric films to monitor in‐ and out‐of‐plane coupled deformations in 3D. The KPM are fabricated by distributing two types of piezoelectric particles (i.e., PZT and BaTiO_3_) in silicon rubber, polarizing and cutting into the kirigami films with regular ligaments. We first calibrate the electrical properties of the piezoelectric films and then investigate the mechanoelectrical response of the KPM under in‐ and out‐of‐plane cyclic loading (i.e., bending and twisting) that leads to stretching and wrinkling of the kirigami ligaments. The experiments are validated with analytical and numerical results and good agreements are obtained in terms of the deformation configurations, stress‐strain, and voltage‐strain responses. The bistable mechanoelectrical response of the KPM is analyzed and the findings indicate that the sensing performance is critically affected by the ligaments. Tailoring the mechanoelectrical response of the PZT films by the mechanical deformation of the kirigami structures, the reported KPM can accurately sense coupled large deformations caused by in‐ and out‐of‐plane deformations with up to 200% strains. In the end, the reported KPM is applied as the ultra‐stretchable sensors to monitor the complex coupled large deformations in the application scenarios of vehicle tire bulge and human posture. The reported KPM opens an exciting venue for controlling mechanical functional metamaterials for active sensing under coupled large deformations in many applications.

## 2 Results

### 2.1 Design and Fabrication of the Kirigami Piezo‐Metamaterials (KPM)

**Figure 1:**
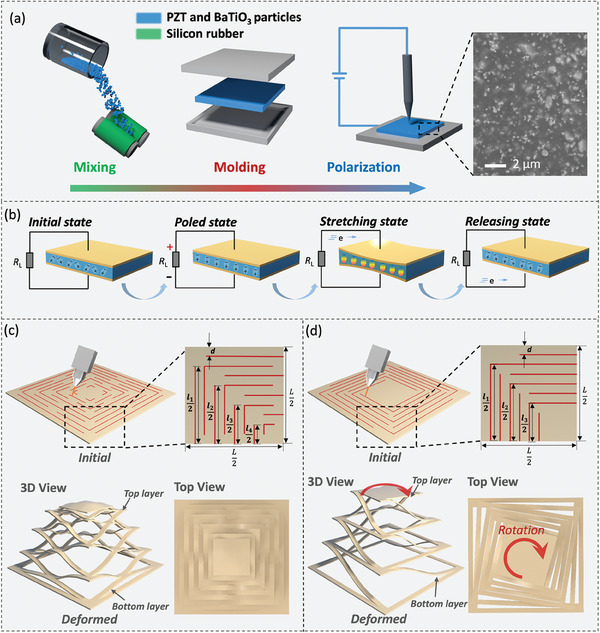
Design and fabrication of the KPM. a) Fabrication process and SEM image of the PZT and BaTiO_3_ rubbery films. b) Mechanism of the PZT and BaTiO_3_ rubbery films in the initial, poled, stretched, and released states. Schematic illustrations of the kirigami patterns and deformation characteristics of the c) C‐KPM and d) R‐KPM.

**Figure** **1** presents the design and fabrication of the KPM. Figure 1a demonstrates the fabrication process of the piezoelectric rubber in the three steps of mixing, molding, and polarization. In the mixing step, the PZT and BaTiO_3_ particles were evenly distributed in the liquid precursor of the ultra‐flexible matrix material (i.e., silicon rubber). Incompatibilities between the microscale particles and polymer precursor led to the aggregation of the particles. We applied shear force and pressure to the rubber using two rollers to redisperse the particles and improve the uniformity of the distribution. After 1 h of mixing, the macroscopic aggregates were completely eliminated and a high percentage (i.e., 80 wt.%) of the piezoelectric ceramic particles were uniformly dispersed in the rubber, which is shown in the scanning electron microscope (SEM) images. The BaTiO_3_‐based KPM was manufactured following the same procedures as the PZT KPM, where the nontoxic BaTiO_3_ particles were used to replace the toxic PZT particles. We particularly measured the density, X‐ray powder diffraction, and high‐resolution scanning electron micrographs of the piezoelectric films to assess the dispersion uniformity of the piezoelectric ceramic particles in the rubbery matrix, as shown in Figures S8 and S9, Supporting Information. In the molding step, the top plate was used to press the composites in the mold into the films with thicknesses of 0.5, 1, and 2 mm. The remaining gas bubbles were removed during this step. The fabrication process of the PZT rubbery films is shown in Video S1 (Supporting Information). In the polarization step, the piezoelectric films were polarized using high‐voltage grid electrodes under an externally applied potential with a field strength of 60 kV cm^−1^ and a temperature of 150 °C for 30 min. The field‐aligned the piezoelectric dipoles in the films along its direction, perpendicular to the planar surface. Details of the fabrication process are provided in Materials and Methods 1 and 2, as well as Figure S1 (Supporting Information).

Figure 1b illustrates the polarization of the piezoelectric films in the initial, poled, stretched, and released states. Initially, the orientation of cations and anions is random and no effective macroscopic polarization exists in the PZT and BaTiO_3_ films. After polarizing, the internal electrical domains are rotated and uniformly aligned following the direction of the polarizing electric field. Lateral stretching by the external force deforms the matrix and rearranges the PZT and BaTiO_3_ particles. This changes the charge balance and causes electrons to flow through the circuit, converting the mechanical work into electrical power. When the force is released, electrons flow back to reach a new equilibrium. Figure 1c,d shows the kirigami structures of the KPM with the centrally and rotationally symmetric patterns.

We design the KPM into the centrally symmetric patterns (C‐KPM) that lead to out‐of‐plane deformation of the central square under pullout displacement.^[^ ^12^ ^]^ Inspired by tetrachiral structures,^[^ ^38^ ^]^ the KPM are also designed into the rotationally symmetric patterns (R‐KPM), which experience rotation of the central square in addition to the out‐of‐plane deformation. The original PZT and BaTiO_3_ films have an elastic modulus of 2.23 MPa and overall sizes of 10 cm × 10 cm × 1 mm and 10 cm × 10 cm × 2 mm. We cut them into the KPM kirigami patterns using a craft cutter. Detailed design parameters are provided in Figure S2 and Table S1 (Supporting Information). The kirigami cutting process is shown in Video S2 (Supporting Information).

### 2.2 Mechanical and Electrical Calibrations of the KPM

**Figure 2:**
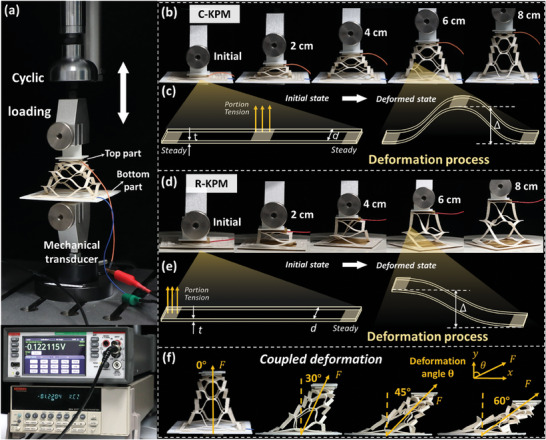
Experimental setup and deformation analysis of the KPM. a) The cyclic tensile testing machine is used to apply the cyclic pullout displacement and record the reaction force, and the electrometer is used to collect the electrical signals generated by the C‐KPM and R‐KPM. b) Different deformation states of the C‐KPM during displacements from 0 to 8 cm. c) Deformation analysis of the symmetric ligaments in the C‐KPM. d) Deformation of the R‐KPM at the same states and the same displacement as the C‐KPM. e) Deformation analysis of the chiral ligaments in the R‐KPM. f) Coupled large deformations of the C‐KPM with the predefined displacement angles of 0°, 30°, 45°, and 60°.

**Figure** **2** presents the experimental setup and deformation analysis of the KPM under cyclic loading. To accurately apply the pullout displacement without tilting or rotating, the KPM was attached to two parallel loading clamps along the outer rims to ensure that they are perpendicular to the displacement, as shown in Figure 2a (also see Video S3, Supporting Information). In addition, the pullout displacement was carefully adjusted to be applied at the centroid of the KPM. The displacement frequency was adjusted from 0 to 5 Hz with an amplitude of up to 10 cm. The mechanical response of reaction force was recorded with the precision of 0.01 N. The voltage output during displacement was measured by the electrometer of Keithley 6514 with low noise (< 1 fA) and high input impedance (> 200 TΩ). Details are provided in Materials and Methods 3. Figure 2b shows the out‐of‐plane deformation of the C‐KPM under pullout displacement at different states, and Figure 2c illustrates the deformation process from the initial to deformed states. Pulling the middle parts of the ligaments causes out‐of‐plane deformations that are evenly distributed over all the symmetric ligaments of the C‐KPM. The out‐of‐plane deformation of the R‐KPM (Figure 2d) was qualitatively different at the same pullout displacement because the chiral ligaments are deformed asymmetrically, as illustrated in Figure 2e. Since application scenarios typically contain coupled deformations due to combined in‐ and out‐of‐plane strains (e.g., tire bulge and human posture in Figure 5), we also consider the coupled large deformations of the KPM with the predefined displacement angles of θ = 0°, 30°, 45°, and 60°, as shown in Figure 2f. The C‐KPM were attached to the parallel clamps with the predefined angles such that the coupled deformations consisted of the in‐ and out‐of‐plane strains, respectively, in the *y* and *x* directions. The experimental setup and loading process are shown in Video S4 (Supporting Information).

### 2.3 Electrical Performance of the KPM

**Figure 3:**
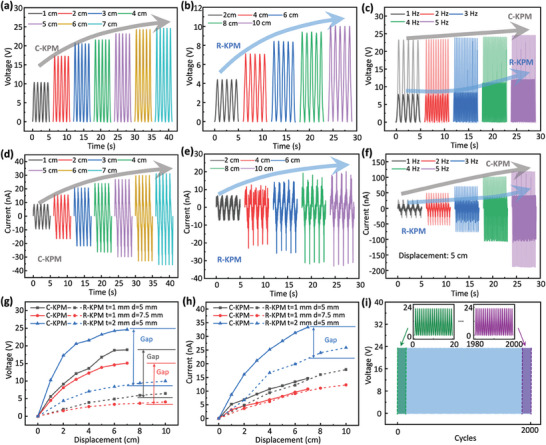
Electrical response of the KPM. Voltage variations of the a) C‐KPM and b) R‐KPM during cyclical 7 and 10 cm displacements at a constant loading frequency of 1 Hz. c) Voltage variations of the C‐KPM and R‐KPM at cyclical displacement of 5 cm at 1–5 Hz. Current variations of the d) C‐KPM and e) R‐KPM at 1 Hz, 7 cm, and 10 cm cyclical displacements. f) Current variations of the C‐KPM and R‐KPM at 1–5 Hz, 5 cm cyclical displacement. g) Peak voltage‐displacement and h) peak current‐displacement relations of the C‐KPM and R‐KPM with different thickness t and width d (θ = 0°). i) Fatigue testing results of voltage for the C‐KPM under the 2000‐cycle out‐of‐plane displacement.

The piezoelectric responses of the KPM are studied under the displacements varied from 1 to 7 cm for the C‐KPM and 2–10 cm for the R‐KPM with the same loading frequencies of 1–5 Hz. **Figure** **3**a,b shows the voltage‐time curves of the C‐KPM and R‐KPM, respectively, with the pullout displacement applied at the center. We first consider the pure pullout displacement at an angle of θ = 0°. The voltages are significantly affected by the out‐of‐plane deformation caused by the displacement. Figure 3c shows the effect of loading frequency on the voltage variations. We observe that the voltage of the C‐KPM is nearly constant under different frequencies, while that of the R‐KPM is increased when the frequency reaches 4 Hz. In the same manner, Figure 3d,e presents the current variations of the C‐KPM and R‐KPM under different displacements, respectively. The currents of the C‐KPM and R‐KPM increase with frequency and saturate at 4 Hz, as shown in Figure 3f. In general, the voltages and currents for all the KPM are changed with frequency between 3 and 4 Hz. We further investigate the roles of the kirigami geometries to the peak voltage and current of the KPM. The thicknesses of *t* = 1 and 2 mm and ligament widths of *d* = 5 and 7.5 mm are particularly considered in Figure 3g,h, respectively, at the displacement angle of 0° and loading frequency of 1 Hz. The voltage and current are proportional to the thickness and inversely proportional to the width of the ligaments. The electrical response of the C‐KPM probably is larger than that of the R‐KPM because the local strain of the C‐KPM is larger than that of the R‐KPM under the same pullout displacement (see Figure 2). Figure 3i illustrates the results of the fatigue testing results of the C‐KPM subjected to cyclic loading. The piezoelectric potentials are observed as a function of the out‐of‐plane displacement, which is fully repeatable up to 2000 cycles at the stretching ratio of 50% and frequency of 1 Hz. See Materials and Methods 4 for details.

### 2.4 Mechanoelectrical Response of the KPM

**Figure 4:**
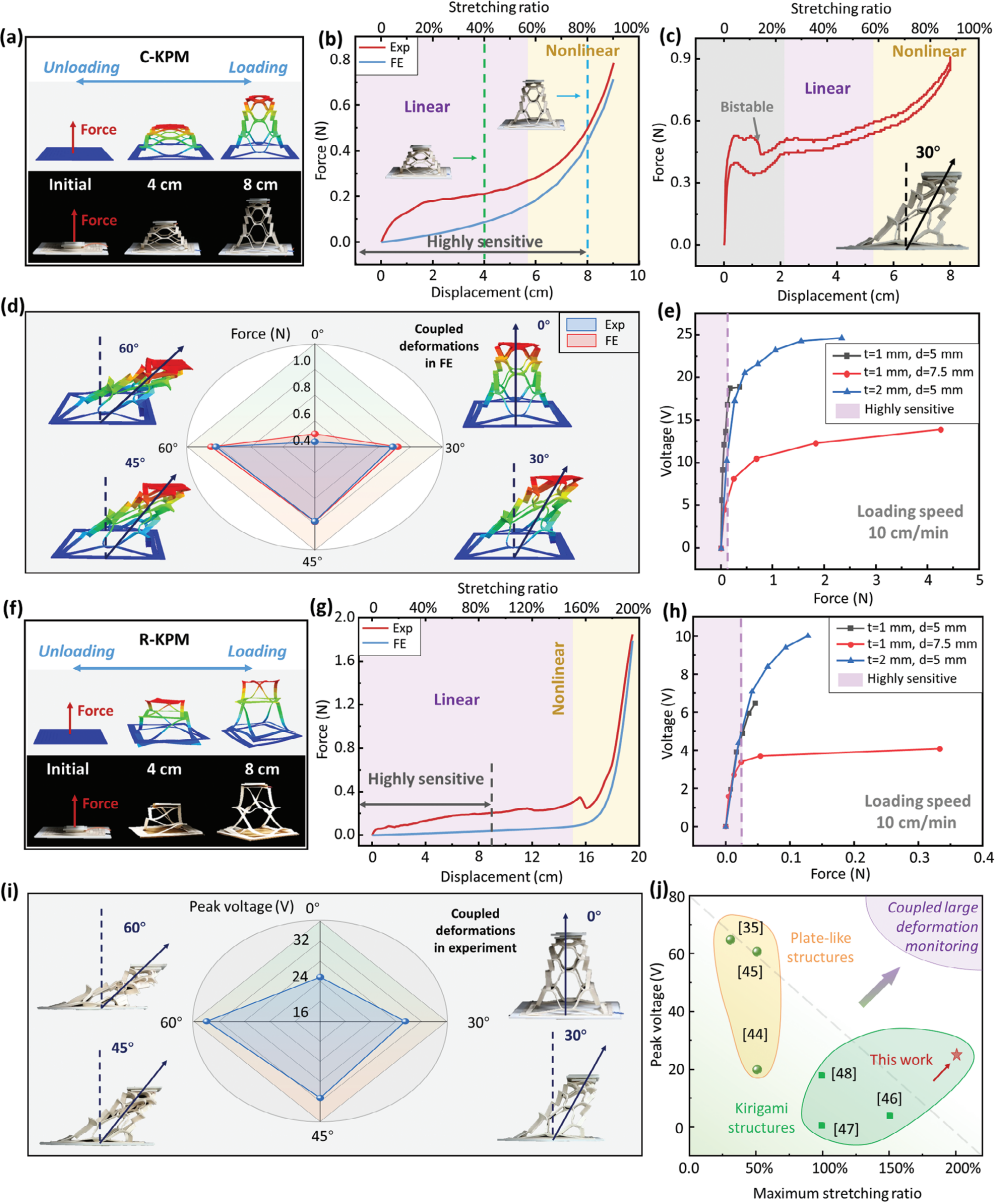
Mechanoelectrical response of the KPM. a) Comparison of the C‐KPM deformation in the FE models and experiments. Force‐displacement relationships of the C‐KPM with b) the thickness of t = 1 mm and ligament width of d = 5 mm and c) at the displacement angle of θ = 30°. d) Comparison of the maximum forces at the displacement of 8 cm from experiments and FE simulations for the C‐KPM under different displacement angles. e) Mechanoelectrical response (i.e., voltage vs force) of the C‐KPM with different thicknesses t and ligament widths d. f) Comparison of the R‐KPM deformation in the FE models and experiments. g) Force‐displacement relationship of the R‐KPM with the thickness of t = 1 mm and ligament width of d = 5 mm. h) Mechanoelectrical response of the same R‐KPM. i) Peak voltages for the C‐KPM under different displacement angles. j) Comparisons of the peak voltage and maximum stretching ratio between the KPM and published piezoelectric films with plate‐like or kirigami structures.^[^ ^34^, ^43^, ^44^, ^45^, ^46^, ^47^ ^]^

**Figure** **4**a displays the out‐of‐plane deformation of the C‐KPM under pullout displacement at θ = 0°, and Figure 4b shows the comparison of the force‐displacement relationships between the experiments and numerical simulations. Numerical models are developed using Abaqus 2016 to investigate the mechanical characteristics (i.e., coupled large deformations and stress‐strain relationship) of the KPM (see Materials and Methods 5 in detail). A nearly linear region of the reaction force is found for the C‐KPM until the pullout displacement reaches 6 cm and then the force enters the nonlinear region with sharply increasing. More results are provided in Figure S3 (Supporting Information). Figure 4c shows the force‐displacement response of the C‐KPM at θ = 30°. A bistable response is observed (in the bistable region) prior to the linearly and nonlinearly increasing regions. This is mainly due to the fact that the C‐KPM experiences in‐ and out‐of‐plane coupled deformations under the pullout testing with the displacement angle. Initially, the in‐plane stretching (with higher deformation resistance) dominates the force‐displacement response, which leads to a sharply increasing force. At a displacement of 5 mm, the deformation mode is changed from in‐ to out‐of‐plane deformations (with lower deformation resistance), which results in the sudden drop of the force response. Similar findings were reported in the earlier study.^[^ ^33^ ^]^ Figure 4d demonstrates the experimental and numerical results of the maximum force with different displacement angles at the pullout displacement of 8 cm for the C‐KPM. Findings indicate that the reaction force increases with the displacement angle because in‐plane deformation plays a more significant role at higher displacement angles with higher deformation resistance, similar to the findings in Figure 4c.

Figure 4e shows the mechanoelectrical responses (i.e., voltage vs force) of the C‐KPM with different thicknesses *t* and ligament widths *d*. The sensing accuracy (i.e., slope) is gradually decreased with the increasing of the force, and therefore, the proposed C‐KPM are more sensitive to the reaction forces less than 0.5 N, which is, 8 cm in pullout displacement as in Figure 4b. Figure 4f displays the out‐of‐plane deformation of the R‐KPM under pullout displacement at θ = 0°, and Figure 4g compares the force‐displacement relationships between the experiments and numerical simulations. In the same manner as the C‐KPM, the reaction force of the R‐KPM is linearly increased until the pullout displacement reaches 15 cm and then sharply increases. Figure 4h shows the mechanoelectrical response (i.e., voltage vs force) of the R‐KPM with different *t* and *d*. Similar findings indicate that the R‐KPM are more sensitive to the reaction forces less than 0.04 N, which is, 9 cm in pullout displacement as in Figure 4g. Figure 4i demonstrates the peak voltage of the C‐KPM under different displacement angles. The maximum voltage is obtained at 60° as the local deformation and stress of the ligaments are increased with the displacement angle. The R‐KPM with a side length of 10 cm can be stretched to the out‐of‐plane displacement of 20 cm without failure, and therefore, the maximum stretching ratio is obtained as 200%. Figure 4j compares the peak voltage of the KPM to the published results on the piezoelectric films with the plate‐like or kirigami structures.^[^ ^34^, ^43^, ^44^, ^45^, ^46^, ^47^ ^]^ The peak voltage of the reported KPM is comparable to the existing materials, but the maximal stretching ratio of 200% is larger than the previously reported 150%.^[^ ^45^ ^]^ A quantitative comparison of the mechanoelectrical performance of the reported KPM and existing piezoelectric materials is provided in Note S9 (Supporting Information), and the stretchability of the KPM is discussed in Note S12 (Supporting Information).

### 2.5 KPM Sensors for Sensing Coupled Large Deformations in Applications

**Figure 5:**
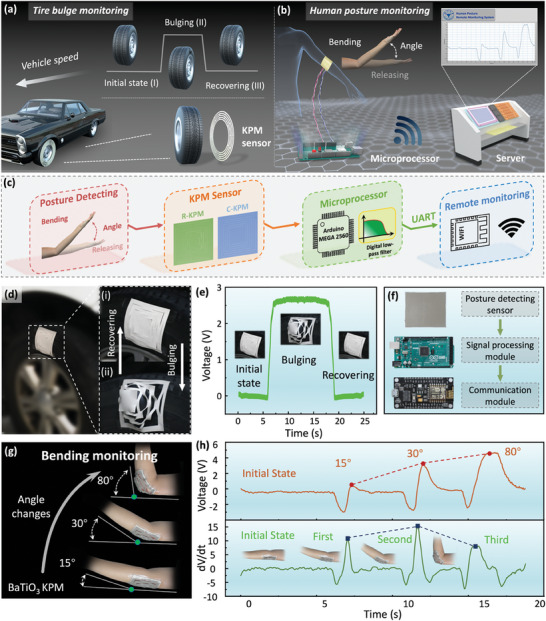
PZT and BaTiO_3_ KPM sensors in active sensing. Schematic application scenarios and working mechanisms of the a) PZT KPM sensors for vehicle tire bulge monitoring and b) BaTiO_3_ KPM sensors in portable wearable devices. c) Schematic diagram of the signal collection and transmission circuit. d) Field‐testing images of the PZT KPM sensors used for tire bulge monitoring. e) Field‐testing results of the voltage curve during the tire bulging deformation. f) Wireless monitoring systems consisted of the sensing module of the KPM sensors, the processing module of Arduino MEGA 2560 and the communication module of ESP8266. g) Nontoxic BaTiO_3_ KPM sensors illustrated in the healthcare applications of human elbow posture monitoring under different bending angles. h) Electrical response of the BaTiO_3_ KPM sensors under different bending angles.

The reported KPM can be used as sensors to accurately monitor coupled large deformations in complex application scenarios such as vehicle tire bulges and human posture. **Figure** **5**a illustrates the vehicle tire bulge monitoring that is a typical scenario involving large in‐ and out‐of‐plane coupled deformations. The PZT KPM sensors can be embedded in the side walls of vehicle tires and triggered by the bulging deformation of the tires at an early stage, hence, the generated electrical signal can be used as a warning for sudden tire bulges. Figure 5b demonstrates the utilization of the BaTiO_3_ KPM sensors in wearable devices for detecting human joint motions, exemplified by the bending and releasing process of a human elbow. To enhance the portability and enable real‐time remote monitoring, the BaTiO_3_ KPM sensors are integrated with a microprocessor system for acquiring voltage signals and wireless transmission to a remote server, as depicted in Figure 5c. Figure 5d presents the PZT KPM sensors bonded on the side walls of the tire for the field testing of the tire bulge. Figure 5e shows the voltage curve generated when inflating the tire. Details on the testing setup are provided in Figure S6 (Supporting Information) and the testing results are shown in Video S5 (Supporting Information). Figure 5f shows the wireless monitoring systems consisting of the sensing module by the KPM sensors, the processing module by Arduino MEGA 2560, and the communication module by ESP8266. Figure 5g,h depicts the deformation and electrical response of the BaTiO_3_ KPM sensors attached to the human elbow with the bending angles of 15^○^, 30^○^, and 80^○^. The experiments indicate that the BaTiO_3_ KPM sensors exhibit more significant integral deformations as the elbow's bending angle increases, which leads to higher output voltage signals. Meanwhile, the positive peaks in the d*V*/d*t* signals can serve as an indicator for the number of elbow bending. The testing results are shown in Video S6 (Supporting Information).

The PZT KPM sensors in this study have certain advantages and drawbacks. PZT has commonly been chosen as the piezoelectric composites with piezoelectric functional phase, which demonstrates remarkable piezoelectric properties and material compatibility.^[^ ^39^ ^]^ In addition, PZT exhibits robust temperature stability and durability, maintaining the piezoelectric performance across a broad temperature range while allowing for operations in varied environmental conditions without significant degradation.^[^ ^40^ ^]^ However, PZT contains lead, which implies severe risks in biomedical or bioengineering applications such as human posture monitoring and raises ecological concerns. Common strategies to address the toxicity of PZT in human‐related applications are separating PZT from human organs^[^ ^41^ ^]^ or replacing PZT with non‐toxic piezoelectric materials.^[^ ^42^ ^]^ Following the first strategy, we have also developed the polyethylene (PE)‐protected PZT KPM by using the medical‐graded, adhesive PE films to sandwich and separate the PZT KPM from directly contacting while tightly bonding to human skins. The sandwiched PE protectors provide necessary protection for PZT KPM sensors in healthcare applications. A detailed description of PE‐protected PZT KPM sensors is illustrated in Note S13 (Supporting Information).

### 2.6 Summary and Conclusion

We report on the ultra‐stretchable kirigami piezo‐metamaterials (KPM) by cutting PZT and BaTiO_3_ rubbery films into the kirigami patterns for sensing in‐ and out‐of‐plane coupled large deformations with up to 200% strains. The KPM was obtained by compounding and polarizing the PZT and BaTiO_3_ particles in silicon rubber and then cutting in the centrally symmetric (C‐KPM) and rotational symmetric patterns (R‐KPM). The C‐KPM was found as the most effective in controlling the electrical performance with the maximum electric response of 24.6 V in voltage and 117.85 nA in charge. Bistable mechanoelectrical responses (i.e., strain‐voltage) were observed for the KPM, which indicated that the output performance can be accurately tailored by adjusting the kirigami structures. Eventually, the PZT KPM sensors were used for wireless monitoring of vehicle tire bulges and the nontoxic BaTiO_3_ KPM sensors were applied for human posture monitoring in healthcare applications. The bulge‐ and posture‐induced voltages of the KPM sensors were accurately detected by the processing module and wirelessly transmitted. This study opens an exciting venue for the mechanical functional metamaterials in active sensing coupled with large deformations for many applications.

## 3 Experimental Section

### Fabrication of the PZT and BaTiO_3_ KPM

The piezoelectric rubbery films were composed of PZT and BaTiO_3_ particles with a diameter of 1 µm (supplied by Quanzhou Qijin New Material and Technology Co., Ltd., China) and raw rubber (supplied by Shenzhen Boruilian Rubber & Plastic Co., Ltd., China) mixed with 5 wt.% curing agents in a weight ratio of 4:1. The mixing was carried out using a roller milling machine with a roll diameter of 10 cm for about 1 h until the particles had been uniformly distributed. The piezoelectric composite was then molded under a pressure of 10 MPa into different thicknesses of 0.5, 1, and 2 mm, respectively. We polarized the resulting PZT‐ and BaTiO_3_‐mixed rubbery films using an external voltage of 60 kV cm^−1^ at 150 °C for 30 min in the air because the elastic matrix tends to absorb oil and swells in an oil bath. The resulting stretchable, piezoelectric films were cut into kirigami patterns using a craft cutter. The fabrication process is illustrated in Figure S1 and Video S1 (Supporting Information).

### Fabrication Conditions Selection for the KPM

*Selection of the Mixing Conditions*: Considering the desired high particle weight ratio of 80%, selecting a suitable mixing instrument and employing an appropriate mixing duration was crucial. The roller milling machine was used to achieve homogeneous mixing over a large pressed area while providing high‐speed full agitation within a certain time period. The piezoelectric particle proportion was carefully tailored to maximize the electromechanical coupling performance of the KPM, as shown in Note S8 (Supporting Information).

*Selection of the Molding Conditions*: Considering the desired piezoelectric film thickness of 0.5, 1, and 2 mm, employing appropriate molding pressure and adequate molding duration was crucial. The preliminary experiments demonstrated that applying the molding pressure of 10 MPa for 24 h can significantly enhance the quality of the piezoelectric films, ensuring complete curing over the entire structures of the KPM.

*Selection of the Polarization Conditions*: According to the preliminary experiments, 60 kV cm^−1^ was selected as the polarization field strength to ensure the optimal ferroelectric polarization effect. The polarization field strength was carefully determined since excessively high field strength would trigger the unstable polarization state (e.g., corona discharge or material breakdown), while insufficient field strength would fail to reach the intended polarization level (i.e., lower d_33_ value).^[^ ^43^ ^]^ 150 °C was selected as the polarization temperature because elevating the polarization temperature can boost the mobility of electric dipoles within the material, consequently increasing the rate of polarization.^[^ ^34^ ^]^ Excessive polarization temperature (e.g., surpassing the Curie temperature) resulted in undesirable material effects such as piezoelectric properties degradation, and low polarization temperature led to a diminished rate of polarization. 30 min was selected as the polarization duration to attain a stable, reproducible polarization state and optimize piezoelectric properties.^[^ ^44^ ^]^ The initial experiments indicated that short time duration led to inadequate polarization (i.e., lower d_33_ value) and long duration did not exert any substantial impact on the polarization effect, as negligible improvements in the d_33_ value.

*Experimental Setup and Testing*: To measure the electrical response of the KPM, two identical wires were connected to the top and bottom of the KPM with insulating tape to prevent short circuits, as shown in Figure 2a. A Keithley System 6514 electrometer was used to record the electrical output, including the open‐circuit voltage V_oc_ and the short‐circuit current I_sc_. A tensile testing machine manufactured by Han Shen Automation (Jinan) Co., Ltd was used to apply cyclic tension on the KPM for the mechanoelectrical response (see Figure 2) in displacement control mode. The edges and central parts of the KPM were stuck to two parallel 3D‐printed PLA plates and connected to the testing machine to characterize the force‐displacement relations, as shown in Figure 4. A professional camera Canon 5D4 was used to record the deformation of the KPM. In the wrist posture monitoring experiment, a processing module with an Arduino MEGA 2560 and a communication module with an ESP8266 were utilized to record voltage signals and transmit them to a server.

*Mechanical Analysis of the KPM*: The loading of the C‐KPM was analogous to an elastic beam with two fixed ends and displacement in the center. The beams of the top three deformation stages can be considered as the beams with fixed ends and displacement was applied to the end parts, while displacement was applied to the middle parts of the bottom ones. Detailed analysis was supplied in Note S3 (Supporting Information). The relationship between the total axial deformation and force can be written as:

(1) $$F=\frac{768EI}{{\left(L_{1}-2w\right)}^{3}+2\left[{\left(L_{2}-2w\right)}^{3}+{\left(L_{3}-2w\right)}^{3}+{\left(L_{4}-2w\right)}^{3}\right]}\;x.$$

For the R‐KPM, as the external force is exerted on the structure, one end of the elastic beams is fixed and the other end deflects in bending. Since the deformation is evenly distributed, all beams are considered as beams with fixed ends and axial displacement is applied to the end parts. The relationship between the total axial deformation and force can be written as:

(2) $$F=\frac{48EI}{{\left(L_{1}-4w\right)}^{3}+{\left(L_{2}-4w\right)}^{3}+{\left(L_{3}-2w\right)}^{3}}\;x.$$

*Numerical Simulations*: The proposed kirigami structures combined with piezoelectric rubber were simulated in Abaqus. The geometric and material properties are listed in **Table** **1**. Due to the complex cutting, both structures were simulated using Tet mesh type and the element type was C3D10. The total face was divided into separate parts using the datum faces. The bottom annular parts of both structures were fixed. A displacement load of 90 mm with constraints of other degrees of freedom was exerted to the middle part of C‐KPM, while a load of 190 mm without any constraint in the x–y plane was applied to the middle part of R‐KPM. The impact of gravity was factored into the total model. Further details about the boundary and loading conditions are provided in Figure S4 (Supporting Information).

**Table 1:** Geometric and material properties of C‐KPM and R‐KPM.

	Factor	Value
Geometric properties (mm)	Side Length *L*	100
	Thickness *t*	1
		2
	Ligament width *d*	5
		7.5
Materials properties	Density (kg mm^−3^)	1.2 × 10^−6^
	Young's modulus (MPa)	2.23
	Poisson's Ratio	0.45

## Conflict of Interest

The authors declare no conflict of interest.

## Supporting information

Supporting Information

Supporting Information

Supporting Information

Supporting Information

Supporting Information

Supporting Information

Supporting Information

Supporting Information

## Data Availability

The data that support the findings of this study are available from the corresponding author upon reasonable request.
