# Supplementary material for: Ultra‐Stretchable Kirigami Piezo‐Metamaterials for Sensing Coupled Large Deformations
Source: Adv Sci (Weinh). 2023 Dec 3;11(5):2303674. doi: 10.1002/advs.202303674 (PMC10837349; doi:10.1002/advs.202303674)
Supplement: Supplementary file 1 — Supporting Information [file ADVS-11-2303674-s001.pdf]

ADVANCED SCIENCE
Open Access

Supporting Information

for *Adv. Sci.*, DOI 10.1002/advs.202303674

Ultra-Stretchable Kirigami Piezo-Metamaterials for Sensing Coupled Large Deformations

Luqin Hong, Hao Zhang, Tobias Kraus and Pengcheng Jiao**

Supporting Information

Ultra-Stretchable Kirigami Piezo-Metamaterials for Sensing Coupled Large Deformations

Luqin Hong[a*], Hao Zhang[a,b*], Tobias Kraus[c,d1], Pengcheng Jiao[a,b1]

[a]: *Institute of Port, Coastal and Offshore Engineering, Ocean College, Zhejiang University, Zhoushan 316021, Zhejiang, China*

[b]: *Engineering Research Center of Oceanic Sensing Technology and Equipment, Zhejiang University, Ministry of Education, China*

[c]: *INM-Leibniz Institute for New Materials, 66123 Saarbrücken, Germany*

[d]: *Colloid and Interface Chemistry, Saarland University, 66123 Saarbrücken, Germany*

* Authors contributed equally to work.

[1] Corresponding authors. Emails: pjiao@zju.edu.cn (P. Jiao) and tobias.kraus@leibniz-inm.de (T. Kraus).

Note S1. Design and Fabrication Process of the piezoelectric rubber

As shown in the Fig S1, the piezoelectric rubber films were composed of PZT particles with a diameter of 1 μm (supplied by Quanzhou Qijin New Material and Technology Co., Ltd., China) and raw rubber mixed with 5 wt % curing agents, in a weight ratio of 4:1. The mixing was carried out using a roller milling machine with a diameter of 10 cm for about 1 hour until uniform distribution has been achieved. Subsequently, the blended piezoelectric composite was subjected to molding under a pressure of 10 MPa for a 24-hour period, resulting in varying thicknesses of 0.5 mm, 1 mm, and 2 mm, respectively. Considering that the elastic matrix tends to absorb oil and swell in oil bath, the resulting piezoelectric films were polarized using an external voltage of 60 kV/cm at 150 °C for 30 mins in air environment. With these steps, stretchable piezoelectric rubber films were successfully fabricated. After measuring, the piezoelectric constant d_{33} of PZT-mixed rubber is 23.4 pC/N. Specific process of PZT-mixed silicon rubber is demonstrated in Video S1.

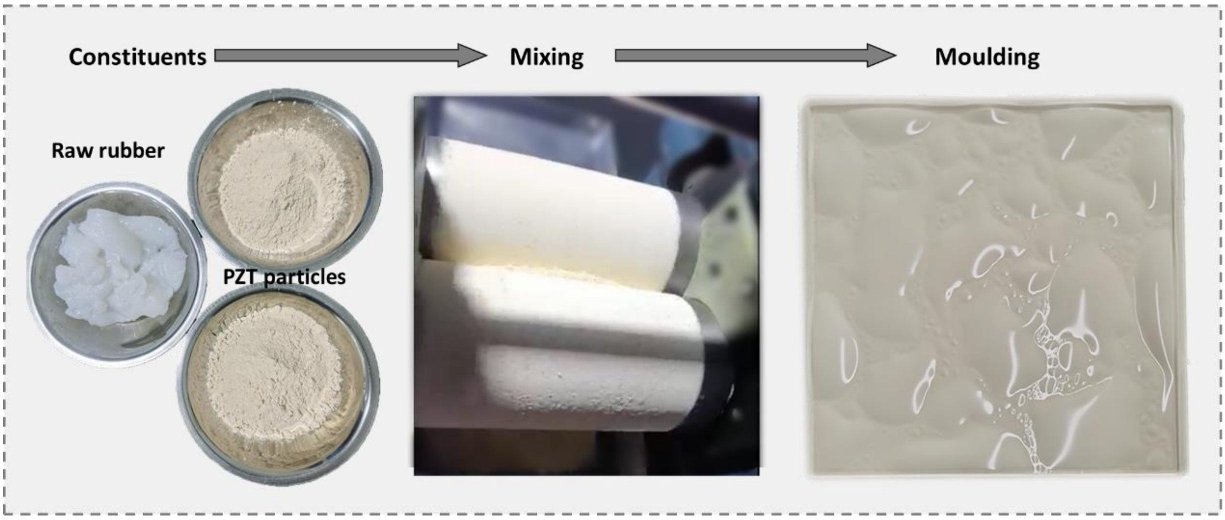

Figure S1. *The fabrication process of the piezoelectric rubber.*

Note S2. Geometric properties of the kirigami structures

Fig S2 shows the design mechanism of KPM sensors. Kirigami patterns were fabricated from PZT mixed piezoelectric silicon rubber plate with an elastic modulus of 2.23 MPa and a total sheet size of $10\ cm \times 10\ cm$. Two kirigami patterns were cut using a craft cutter and three sizes were considered. The first design is center symmetric. While the other one is rotational symmetric. The fabrication process of kirigami patterns on piezoelectric plate is shown in Video S2. At this moment, the KPM sensors preparation is completed.

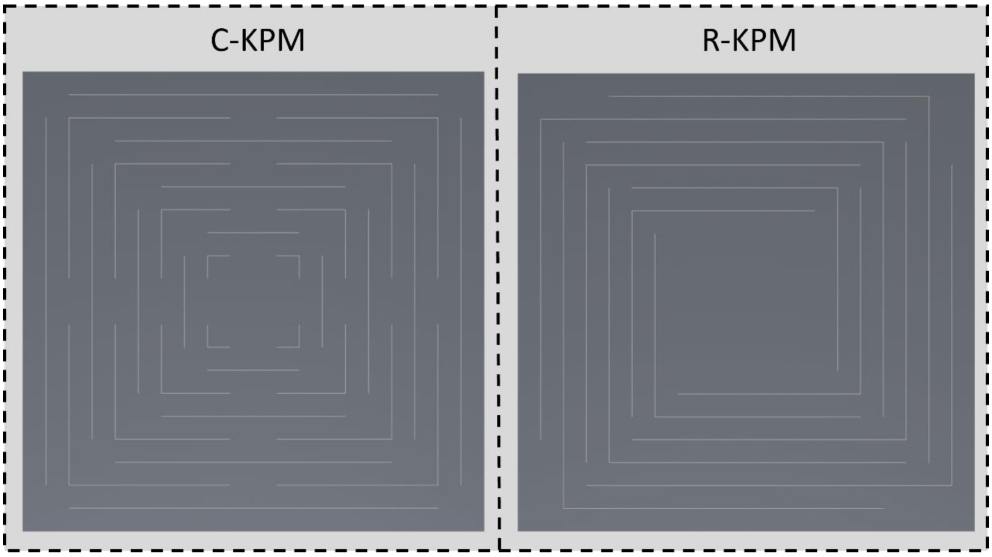

Figure S2. *The design mechanism of the KPM sensors.*

To study the effect of thickness t, width d of ligaments, different parameters are designed. Table S1 presents the geometric properties of two kinds of kirigami structures, including the total length L_1, total width L_2, and thickness t, width d of ligaments.

Table S1. *Design parameters of kirigami structures.*

<table>
<tr><th colspan="2">Fixed factors</th><th colspan="6">Variables</th></tr>
<tr><td>Length L_1 (mm)</td><td>100</td><td rowspan="2">Design</td><td>Center-symmetric structure (C-KPM)</td><td rowspan="2">Thickness t (mm)</td><td>1</td><td rowspan="2">Width of ligaments d (mm)</td><td>5</td></tr>
<tr><td>Width L_2 (mm)</td><td>100</td><td>Rotational-symmetric structure (R-KPM)</td><td>2</td><td>7.5</td></tr>
</table>

Note S3. More mechanical results of KPM sensors

For the C-KPM, the top three layers are symmetric in the vertical direction while the bottom layer is single. Therefore, the total axial deformation can be written as:

$$x = \Delta_1 + 2(\Delta_2 + \Delta_3 + \Delta_4), \tag{1}$$

where Δ_i is the deformation of each layer. When analyzing the elongation of the ligaments, the deformation caused by the shear force is negligible. Since the external force is exerted to the C-KPM, the two ends of the elastic beams are fixed and the middle is stretched. The beams of the top three layers can be considered as the beams with fixed ends and displacement is applied to the end parts, while displacement is applied to the middle parts of the bottom ones. The total force is then divided into four equal parts for each beam in each layer, and therefore, the relationship between the displacement and force can be written as

$$\Delta_1 = \frac{Fl_1^3}{768EI} \tag{2}$$

and

$$\Delta_i = \frac{Fl_i^3}{384EI} \ (i = 2,3,4). \tag{3}$$

Substituting Eqs. (2) and (3) into Eq. (1) yields the relationship for the C-KPM as

$$F = \frac{768EI}{l_1^3 + 4l_2^3 + 4l_3^3 + 4l_4^3} x, \tag{4}$$

where the length of the ligaments l_i is related to the width d as

$$l_i = L - 4id \ (i = 1,2,3,4) \tag{5}$$

For the R-KPM, different from the C-KPM, there are only three layers. Therefore, the total axial deformation can be written as

$$x = \Delta_1 + \Delta_2 + \Delta_3. \tag{6}$$

As the external force is exerted to the structure, one end of the elastic beams is fixed and the other end deflects in bending. Since the deformation is evenly distributed to each layer, all beams are considered as the beams with fixed ends and axial displacement is applied to the end parts. The total force is divided into four equal parts for each beam in each layer. Therefore, the relationship between the displacement and force can be written as

$$\Delta_i = \frac{FL_i^3}{48EI} \ (i = 1,2,3). \tag{7}$$

Substituting Eq. (7) into Eq. (6) yields the relationship for the R-KPM as

$$F = \frac{48EI}{l_1^3 + l_2^3 + l_3^3} x. \tag{8}$$

Note S4. More mechanical results of KPM sensors

Figure S3 displays more results of Force-Displacement relationship of C-KPM and R-KPM with different design parameters. There exist similar variation trends for 3 designs of C-KPM and 3 designs of R-KPM, respectively. There are also deviations between experimental and numerical results. In simulation, the contacts between element faces are not considered. For C-KPM, the friction between adjacent ligaments (see Figure. 2(c)) is distinct when the reaction force is small at the beginning loading process. With the increasing of deformation, the upper ligament parts are independent and there exists no contact with each other, so the numerical result agrees well with experimental result during the large deformation process. For R-KPM, the friction is only from one side of ligaments (see Figure. 2(e)) and nearly half of C-KPM, on account of which these two results have better accordance.

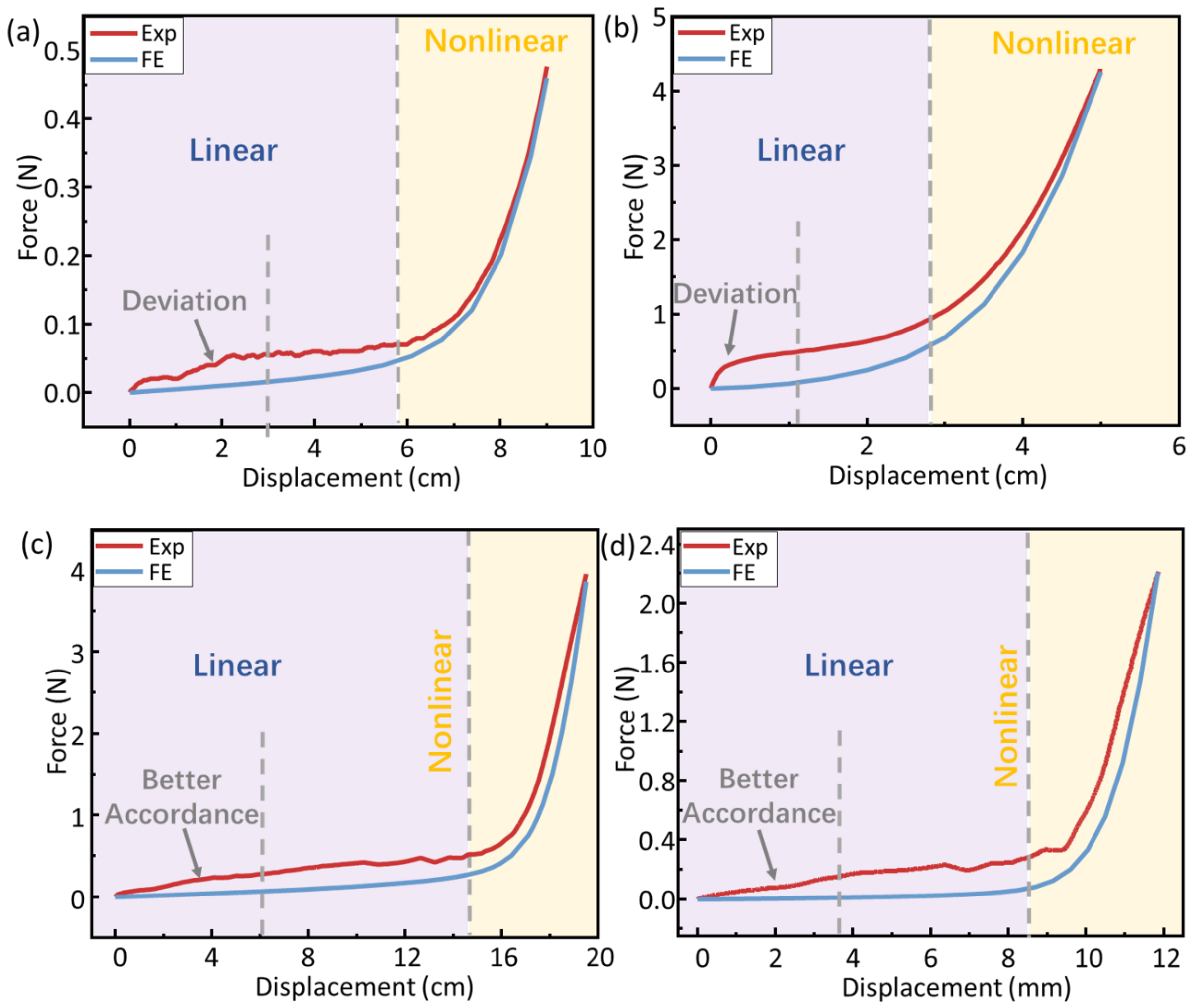

Figure S3. *More mechanical results of KPM sensors. C-KPM: (a) t=0.5 mm, d=5 mm; (b) t=1mm, d=7.5 mm; R-KPM: (c) t=2 mm, d=5 mm (d) t=1mm, d=7.5 mm.*

Note S5. Numerical Simulations of the KPM sensors

The proposed kirigami structures combined with piezoelectric hyperelastic materials are simulated in ABAQUS. The materials properties are listed in Table S2. The two structures were simulated with Tet mesh type due to the complex cutting. To carry out the deformation of the KPM sensors, the total face was separated into different parts by the datum face. The bottom annular parts of both structures were fixed and 90 mm displacement load was exerted to the top parts with constraint in x-y plane for C-KPM, while 190 mm displacement load was exerted to the top parts with no constraint in x-y plane for R-KPM. Taking the reality into account, the gravity is imposed to the total model. The boundary and loading conditions are provided in Fig S4.

Table S2*. material properties of piezoelectric silicon rubber.*

Silicon materials	Density (kg/mm3)	1.2×10^{-6}
	Young's modulus (MPa)	2.23
	Poisson's Ratio	0.45

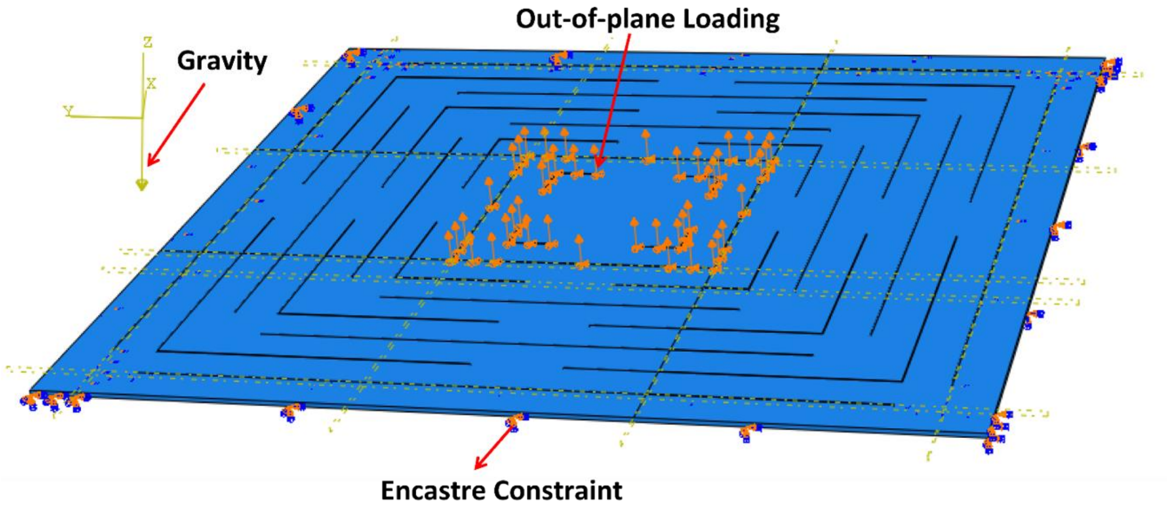

Figure S4. Load and boundary conditions of the KPM sensors.

Note S6. Cyclic loading and unloading tests of KPM structures

Fig. S5 demonstrates the cyclic loading and unloading tests of KPM structures (set $t = 2\,mm, d = 5\,mm$ as example), which shows good stability from mechanical perspective. Additionally, the area difference in the graph represents the dissipated energy during the loading and unloading process.

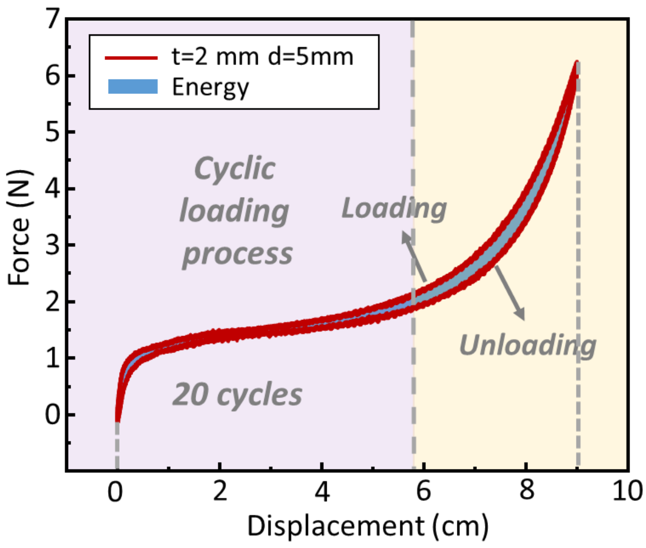

Figure S5. Force-displacement relationship of the C-KPM subjected to the cyclic loading with 20 cycles.

Note S7. Field testing setup of KPM sensors

Figure S6 shows the field testing setup. Wireless power was used to power the electrometer and the laptop was used to collect the data from the electrometer, including open-circuit voltage V_{oc} and short-circuit current I_{sc}. To monitor the state of the tire, the KPM configuration as an active sensor was glued to the air balloon in the surface of the tire. The air balloon was inflated by air pump to simulate the tire bulge motion.

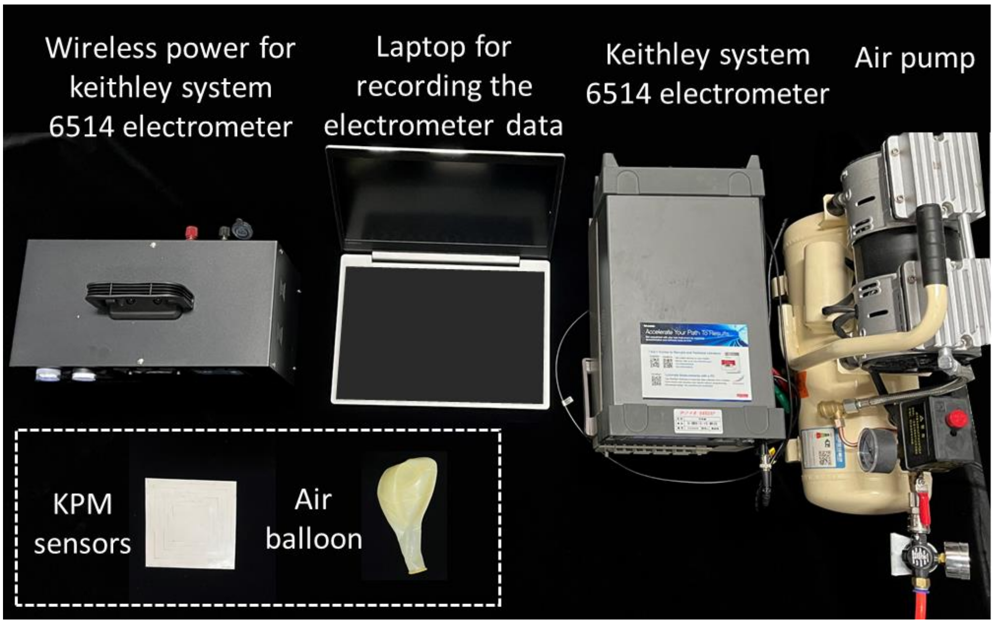

Figure S6. *Field testing setup of applying KPM sensors glued to the surface of the tire to monitor the state of the tire.*

Note S8. PZT proportion selection for the KPM

Figure S7 shows the influence of the PZT proportion on the coupling electromechanical performance of the piezoelectric film samples [35]. Increasing the PZT particle weight ratio enhances the electrical performance of the piezoelectric samples while decreasing their mechanical properties. According to our preliminary testing, the optimal PZT particle weight ratio was found as 80 wt% since this configuration led to the optimal coupling electromechanical performance simultaneously. Satisfying the mechanical performance requirements (i.e., maximum stretching ratio: 200%), we selected the PZT proportion that the electrical performance reached its peak, ss shown in Figure S7.

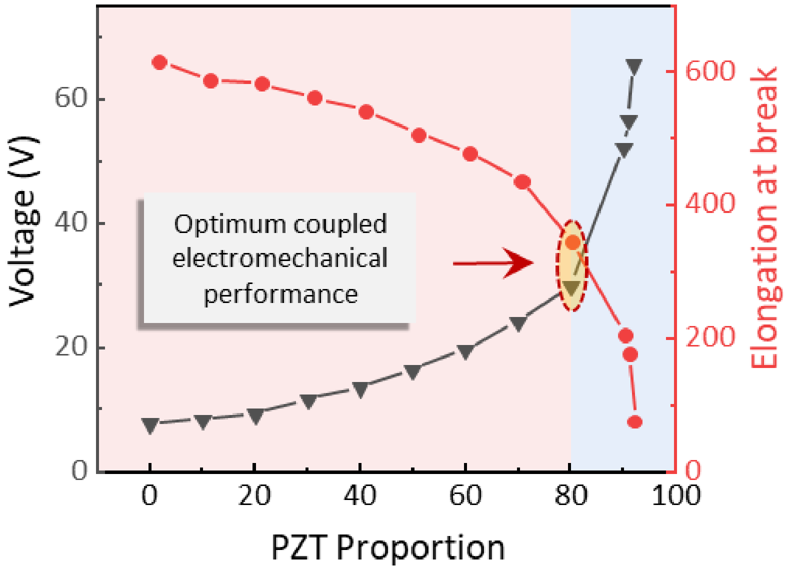

Figure S7. *The influence of PZT proportion on the coupling electromechanical performance of piezoelectric composite materials [35].*

Note S9. Comparation of the mechanoelectrical performance between the reported KPM sensors and the existing piezoelectric sensors

Figure 4(j) and Table S3 compare the mechanoelectrical performance (i.e., maximum stretching ratio and peak voltage) of our KPM sensors and previous reports. Our design makes progress in three-dimensional coupled in- and out-of-plane deformations monitoring of complex applications. Comparing with other plate-like sensors in the existing studies, the reported KPM sensors exhibit notable mechanical superiority, especially for monitoring coupled large deformations. Such advantage enhances their applicability in the scenarios requiring substantial deformations, which expands their potential applications. In addition, we have compared the deformation configurations of the KPM sensors with the existing piezoelectric sensors. The KPM sensors take advantage of the kirigami structures to significantly expand the stretching range and direction of the existing flexible PZT films. The peak voltage of the reported KPM is comparable to the existing materials, but the maximal stretching ratio of 200% is larger than the previously reported 150% [46]. The distributions of the peak voltages under different displacement angles are depicted in Figure 4(i).

Table S3. *Comparation of the structural design, material composition, and mechanoelectrical performance between the reported KPM sensors and the existing piezoelectric sensors.*

Stretchable structures	Piezoelectric materials	Substrate	Maximum stretching ratio	Peak voltage	References
Plate-like structure	PZT	Solid silicon rubber	30%	65 V	Ref. [35]
Plate-like structure	PZT	Solid silicon rubber	50%	61 V	Ref. [45]
Plate-like structure	PZT	Solid silicon rubber	50%	25 V	Ref. [44]
Kirigami structure	PVDF	--	150%	1.2 V	Ref. [46]
Kirigami structure	$BaTiO_3$	PDMS	100%	0.8 V	Ref. [47]
Kirigami structure	PZT	PDMS	100%	17.5 V	Ref. [48]
Kirigami structure	PZT	Solid silicon rubber	200%	24.6V	This work

Note S10. Density measurement for three piezoelectric film samples

A MH-300A densitometer with a ±0.02 g/cm^3 margin of error was selected to quantify the density of three piezoelectric film samples with the same length and thickness but different widths, as shown in Figures S8(a) and (b). The density and mass distributions of the three samples are shown in Figure S8(c). Two samples had the same density of 3.495 g/cm^3 and the third sample has the density of 3.465 g/cm^3. These results indicate the uniform density distribution of the piezoelectric film samples with density differences below 1%. The density measurement procedures for the piezoelectric film samples are observed in Supporting Video S7.

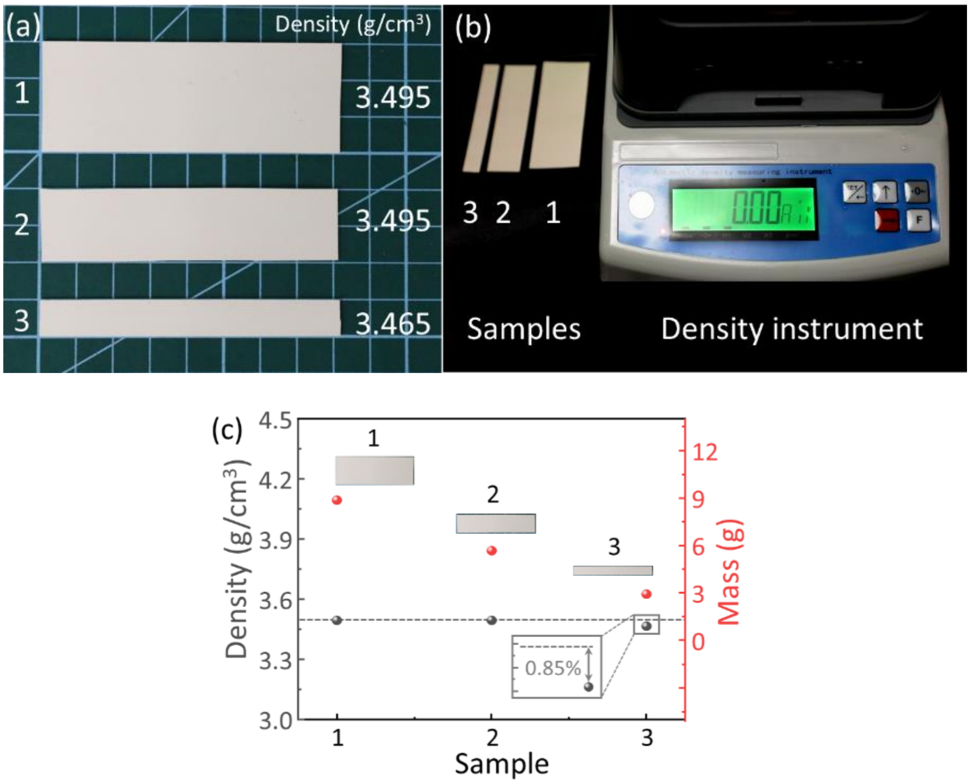

Figure S8. Density measurement for three PZT piezoelectric film samples. *(a) Three piezoelectric film samples with the same length and thickness but different widths. (b) Photographs of the measuring instruments and three samples. (c) Density and mass distributions of the three piezoelectric film samples.*

Note S11. X-ray diffraction comparison and high-resolution SEM images of the PZT rubbery films

We used an XPert3 Powder X-ray diffractometer with Cu X-ray radiation operating at 40 kV and 40 mA, covering a reflection angle range (2θ) of 10°-80°. The three XRD patterns showed identical positions and intensities of crystal peaks, as shown in Figure S9(a). Figure S9(b) illustrates the content of substance components comparison between three positions in a KPM sample, as determined by XRD patterns. We conclude that the sample is uniform with the maximum difference in substance components of 3.4%, which indicates a uniform dispersion of the piezoelectric particles (i.e., PZT and $BaTiO_3$) in the matrix. Besides, the high-resolution SEM images of the PZT rubbery films was added to illustrates the uniform dispersion of the piezoelectric ceramic particles in the rubber matrix, as shown in Figure S9(c).

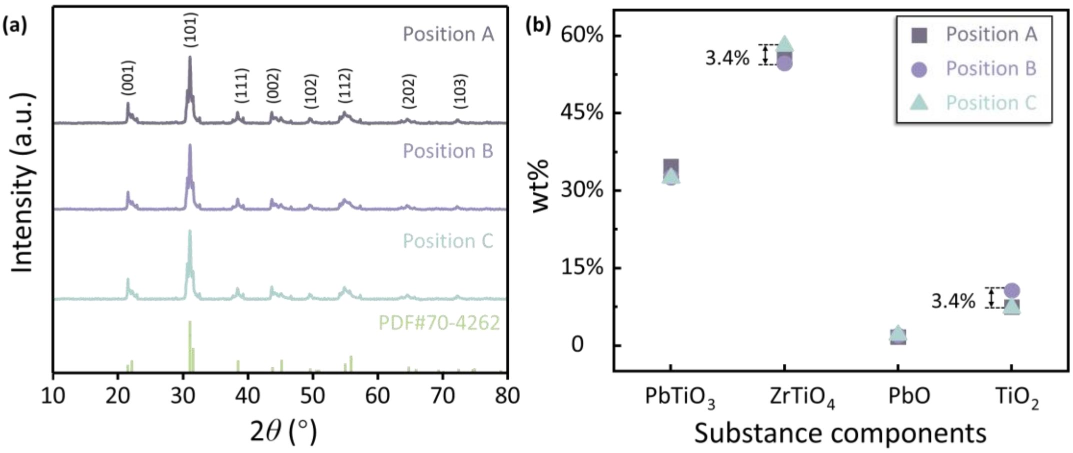

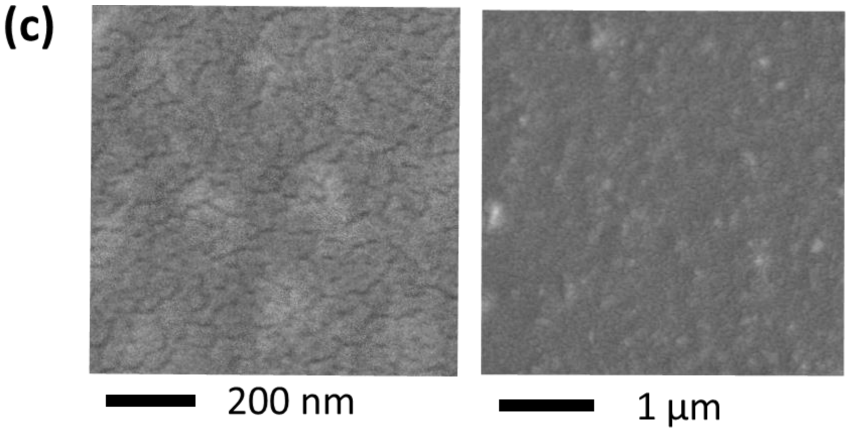

Figure S9. X-ray diffraction comparison and high-resolution SEM images of the PZT rubbery films*. (a) X-ray diffraction (XRD) comparison between three positions in a KPM sample. (b) Content of substance components comparison between three positions in a KPM sample. (c) High-resolution SEM images of the PZT rubbery films.*

Note S12. Illustration of stretchability of the KPM

In this study, the stretchability of the KPM is measured by its stretching ratio. Figure S10(a) schematically illustrates the out-of-plane deformation the R-KPM. The stretching ratio of the R-KPM is defined as $\varepsilon = \frac{H}{L}$, where H is the maximum stretching height and L is the side length. Notably, the R-KPM with the side length of 10 cm can be stretched to the out-of-plane displacement of 20 cm without failure, and therefore, the maximum stretching ratio is obtained as 200%. Therefore, the maximum stretching ratio is obtained as 200%, as shown in Figure S10(b).

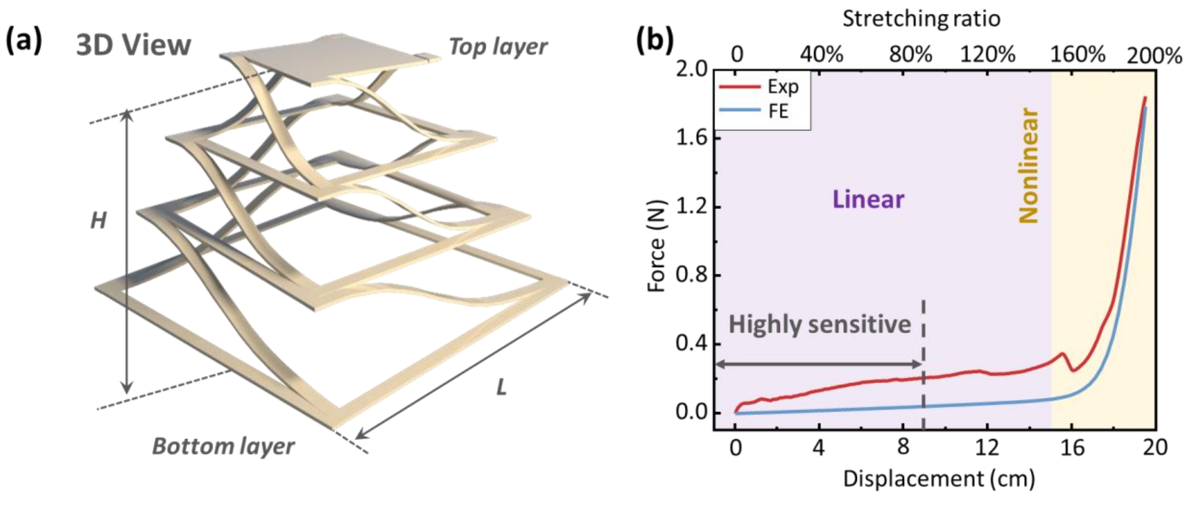

Figure S10. Illustration of stretchability of the KPM. *(a) Schematic illustrations of the stretching (i.e., out-of-plane deformation) of the R-KPM. (b) Force-displacement relationship of the R-KPM under the out-of-plane loading displacement of 20 cm.*

Note S13. PE-protected PZT KPM

The potential toxic risk of the PZT KPM can be effectively mitigated by separating the contact surfaces using barrier materials. To this end, we used the ultrathin medical-graded polyethylene (PE) films to avoid the PZT KPM from directly

contacting human skins, such that to provide necessary protection for human in healthcare applications. Figure S11(a) demonstrates the PZT KPM with the adhesive PE films on the top and bottom surfaces in the application of human wrist posture monitoring. The PZT KPM are sandwiched between the two layers of the PE films and bonded on the wrist, thereby ensuring the deformations together with the human wrist while preventing direct skin contacts. Figure S11(b) shows the field-testing images of the PE-protected PZT KPM sensors attached to the human wrist under different postures (i.e., bending and releasing states).

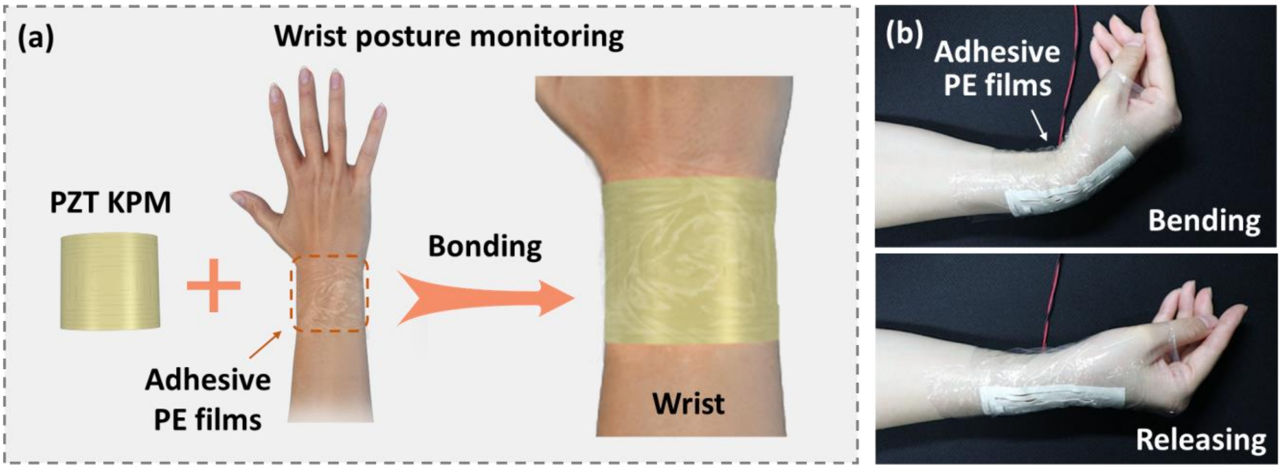

Figure S11. PE-protected PZT KPM. *(a) Separation of the toxic PZT KPM by adhesive PE films for human wrist posture monitoring. (b) Field-testing images of the PE-protected PZT KPM sensors attached to the human wrist under different postures (i.e., bending and releasing states).*

References (As the order in the revised manuscript)

[35] Niu, X., Jia, W., Qian, S., Zhu, J., Zhang, J., Hou, X., ... & Chou, X. (2018). High-performance PZT-based stretchable piezoelectric nanogenerator. ACS Sustainable Chemistry & Engineering, 7(1), 979-985.

[44] Chou, X., Zhu, J., Qian, S., Niu, X., Qian, J., Hou, X., ... & Xue, C. (2018). All-in-one filler-elastomer-based high-performance stretchable piezoelectric nanogenerator for kinetic energy harvesting and self-powered motion monitoring. Nano Energy, 53, 550-558.

[45] Zhu, J., Qian, J., Hou, X., He, J., Niu, X., Geng, W., ... & Chou, X. (2019). High-performance stretchable PZT particles/Cu@ Ag branch nanofibers composite piezoelectric nanogenerator for self-powered body motion monitoring. Smart Materials and Structures, 28(9), 095014.

[46] Kim, Y. G., Hong, S., Hwang, B., Ahn, S. H., & Song, J. H. (2022). Improved performance of stretchable piezoelectric energy harvester based on stress rearrangement. Scientific Reports, 12(1), 19149.

[47] Peng, Y., Li, Y., & Yu, W. (2022). Kirigami‐Based Flexible, High‐Performance Piezoelectric/Triboelectric Hybrid Nanogenerator for Mechanical Energy Harvesting and Multifunctional Self‐Powered Sensing. Energy Technology, 10(8), 2200372.

[48] Hong, Y., Wang, B., Lin, W., Jin, L., Liu, S., Luo, X., ... & Yang, Z. (2021). Highly anisotropic and flexible piezoceramic kirigami for preventing joint disorders. Science advances, 7(11), eabf0795.
